# Simulated effect of calcification feedback on atmospheric CO_2_ and ocean acidification

**DOI:** 10.1038/srep20284

**Published:** 2016-02-03

**Authors:** Han Zhang, Long Cao

**Affiliations:** 1 School of Earth Sciences, Zhejiang University, Hangzhou, Zhejiang 310027, China; 2 State Key Laboratory of Satellite Ocean Environment Dynamics, Second Institute of Oceanography, Hangzhou, Zhejiang, China

## Abstract

Ocean uptake of anthropogenic CO_2_ reduces pH and saturation state of calcium carbonate materials of seawater, which could reduce the calcification rate of some marine organisms, triggering a negative feedback on the growth of atmospheric CO_2_. We quantify the effect of this CO_2_-calcification feedback by conducting a series of Earth system model simulations that incorporate different parameterization schemes describing the dependence of calcification rate on saturation state of CaCO_3_. In a scenario with SRES A2 CO_2_ emission until 2100 and zero emission afterwards, by year 3500, in the simulation without CO_2_-calcification feedback, model projects an accumulated ocean CO_2_ uptake of 1462 PgC, atmospheric CO_2_ of 612 ppm, and surface pH of 7.9. Inclusion of CO_2_-calcification feedback increases ocean CO_2_ uptake by 9 to 285 PgC, reduces atmospheric CO_2_ by 4 to 70 ppm, and mitigates the reduction in surface pH by 0.003 to 0.06, depending on the form of parameterization scheme used. It is also found that the effect of CO_2_-calcification feedback on ocean carbon uptake is comparable and could be much larger than the effect from CO_2_-induced warming. Our results highlight the potentially important role CO_2_-calcification feedback plays in ocean carbon cycle and projections of future atmospheric CO_2_ concentrations.

Atmospheric CO_2_ concentration has increased by 40% since the preindustrial time^1^ primarily due to human activities of fossil fuel burning and deforestation. Between 1959 and 2011 total anthropogenic CO_2_ emission is 436 PgC (1PgC = 10^15^ gram carbon = 1 billion ton carbon) with 44% of the emission stayed in the atmosphere, while 29% and 27% of emissions were absorbed by the terrestrial biosphere and the ocean, respectively^2^. Increasing atmospheric CO_2_ warms the Earth by trapping long wave radiation. In addition, the oceanic uptake of anthropogenic CO_2_ perturbs ocean chemistry by making the ocean more acidic, a process known as ocean acidification^3^.

Global warming and ocean acidification would influence many processes of the marine carbon cycle, which in turn affect atmospheric CO_2_ and climate change. For instance, rising atmospheric CO_2_ concentration leads to an increase of sea surface temperature, reducing CO_2_ solubility in the ocean. As a result, a warmer ocean reduces the oceanic CO_2_ uptake, which provides a positive feedback to the growth of atmospheric CO_2_^4^,^5^. A warmer ocean could also accelerate respiration/remineralization rate of organic carbon and cause a reduction in the vertical flux of particulate organic carbon (POC) to the abyssal ocean. This reduced vertical transport of POC would weaken the ocean biological pump and decrease the ocean’s ability to take up carbon, providing a positive feedback to the growth of atmospheric CO_2_^6^. On the other hand, increased C:N:P stoichiometry in seawater due to rising partial pressure of CO_2_ in the ocean could enhance extracellular organic matter production, strengthening the ocean biological carbon pump and providing a negative feedback to rising atmospheric CO_2_^7^,^8^,^9^.

In addition to global warming, ocean acidification, through its effect on the ocean carbon cycle, could provide feedbacks to atmospheric CO_2_. Global mean ocean surface pH, which can be used to quantify the degree of ocean acidification, has dropped by 0.1 units^1^, representing a 26% increase in hydrogen ion concentration since the industrial revolution. The rise of hydrogen ion concentration would consequently lower carbonate ion concentration 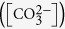 and in turn causes a reduction of seawater CaCO_3_ (aragonite or calcite) saturation state, which is defined as


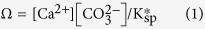


Here, 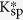 is the stoichiometric solubility product for aragonite or calcite, which are two different polymorphs of CaCO_3_^10^.

Calcifying organisms that use CaCO_3_ to precipitate their shells or skeletons may not be able to acclimate to the reduction of CaCO_3_ saturation state^11^. Gattuso *et al.* detected a nonlinear relationship between calcification rate and CaCO_3_ saturation state from experimental results of certain types of coral species, and suggested calcification rate may drop substantially as a result of decreasing aragonite saturation state^12^. Langdon *et al.* through mesocosm experiment results, argued that declining aragonite saturation state is a primary factor that attenuates coral reef calcification^11^. Laboratory experiments with coccolithophorids^13^,^14^ and analyses of foraminiferal shell weight observational record across glacial-interglacial Termination^15^ indicated a reduction in CaCO_3_ production with increasing CO_2_ concentration and resulting ocean acidification. More recent observational or experiment results also provided evidences of the negative response of calcification to ocean acidification^16^,^17^,^18^. In spite of the abundant observational and experimental evidence, the sensitivity of the response of calcification to acidification varies dramatically between experiments using different species of calcifying groups or manipulation methods.

The process of calcification decreases 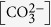, suppressing the ocean’s ability to absorb atmospheric CO_2_. Therefore, the potential reduction of calcification as a result of CO_2_-induced ocean acidification could enhance the ocean’s uptake of carbon, providing a negative feedback for rising atmospheric CO_2_^19^, which is termed as CO_2_-calcification feedback here. Recently, a few modeling studies have been conducted to examine effects of the CO_2_-calcification feedback. In these studies, the parameterization schemes that link CaCO_3_ production to CaCO_3_ saturation state (Ω) are based on different results of experimental studies, and as a consequence, estimates of the effect on oceanic uptake of atmospheric CO_2_ from CO_2_-calcification feedback varies among studies^20^,^21^,^22^,^23^,^24^.

As an extension of previous studies, here we further examine the effect of CO_2_-calcification feedback on the oceanic uptake of atmospheric CO_2_. We incorporate the calcification-Ω dependence into an Earth system model of intermediate complexity to quantify the strength of the CO_2_-calcification feedback in mitigating the growth of atmospheric CO_2_ and ocean acidification. Usually, previous studies on CO_2_-calcification feedback assume a single type of calcification-Ω parameterization scheme. Here, different types of calcification-Ω parameterization schemes are used, which enables us to assess the importance in the parameter value (parameter uncertainty) and the equation form (model structural uncertainty) of the calcification response to ocean acidification. Also, we compare the strength of CO_2_-calcification feedback with the feedback induced by climate change. Furthermore, we assess the effect of different parameterization of CO_2_-calcification feedback on projected ocean acidification. This study aims to further our understanding in the role of the CO_2_-calcification feedback on the ocean carbon cycle and atmospheric CO_2_, which is important for a reliable projection of future atmospheric CO_2_ concentrations and climate change.

## Results

**Figure 1:**
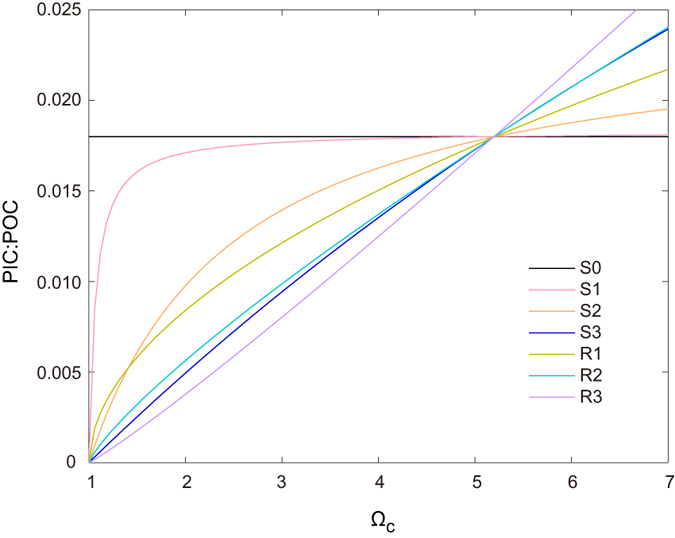
Dependence of CaCO_3_: POC production ratio 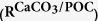 on 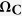 (CaCO_3_ saturation state) used in model parameterizations. Detailed configuration of different model versions is provided in Table 1.

To quantify the effect of CO_2_-calcification feedback, we conduct a series of Earth system model simulations that incorporate different parameterization schemes representing the dependence of calcification rate on saturation state of CaCO_3_ (refer to Method section and Fig. 1). All model simulations last from year 1800 to 3500 with SRES A2 CO_2_ emission scenario until year 2100 and zero CO_2_ emission afterwards. Total cumulative anthropogenic CO_2_ emissions amount to 2270 PgC.

To quantify the effect of climate change on the carbon cycle, we also conduct additional simulation experiments in which CO_2_-induced warming does not affect the carbon cycle. A detailed description of the model and simulation experiments can be found in the Method section.

### Model-observation comparison

**Figure 2:**
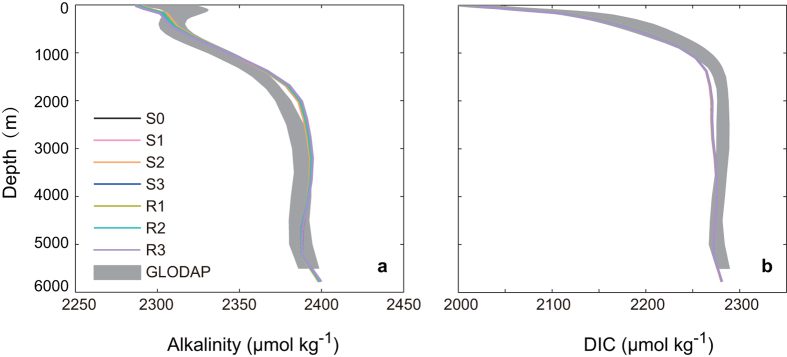
Model-simulated global mean vertical distribution of dissolved inorganic carbon (DIC) and alkalinity (ALK) in mid 1990s compared with observed GLODAP data (including published errors as gray shaded areas)^25^. Model results are shown for different parameterization schemes of CO_2_-calcification feedbacks. Detailed configuration of different model versions is provided in Table 1.

To test the performance of the UVic model in simulating present day carbon cycle, the model-simulated distributions of key variables of the ocean carbon cycle are compared with GLODAP observations^25^. As shown in Fig. 2, the simulated vertical profiles of alkalinity and dissolved inorganic carbon (DIC) are in close agreement with observational estimates, and their vertical profiles of different model versions are almost indistinguishable. This is because in different model parameterizations, 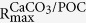 in equation (3) and 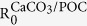 in equation (4) was back-calculated to ensure that the global mean value of 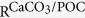 at preindustrial time is the same for all model versions.

In the simulations with climate change modeled CaCO_3_ productions with different parameterizations of 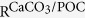 in year 2000 range from 0.69–0.73 PgC yr^−1^, which are within the estimate range of 0.6–1.6 PgC yr^−1^ calculated from observations of satellite and sediment trap^20^.

### Future projections

In the following, we first present results from simulations including the effect of CO_2_-induced warming on the ocean carbon cycle. Then we compare the effect of CO_2_-calcification feedback with that from CO_2_-induced warming, which is obtained by the differences between the simulations with and without CO_2_-induced warming.

**Figure 3:**
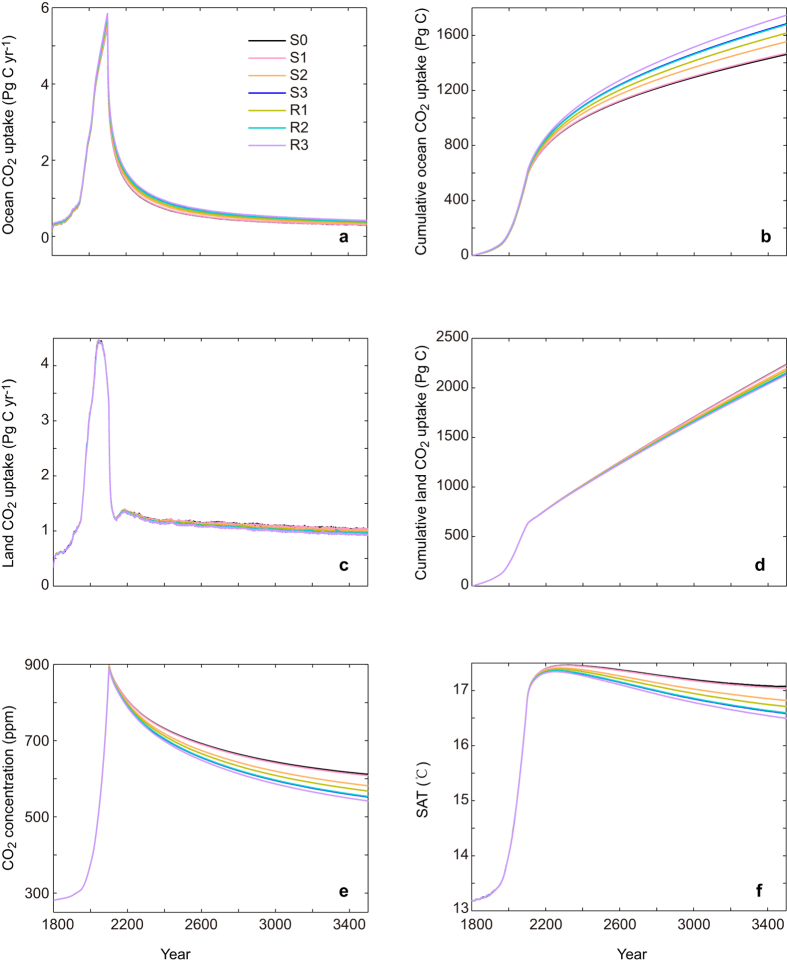
Model-simulated time series of annual and global mean variables for (a) ocean CO_2_ uptake, (b) cumulative ocean CO_2_ uptake, (c) land CO_2_ uptake, (d) cumulative land CO_2_ uptake (e) atmospheric CO_2_ concentration, (f) surface air temperature (SAT). Model results are shown for different parameterization schemes of CO_2_-calcification feedbacks. Detailed configuration of different model versions is provided in Table 1.

**Figure 4:**
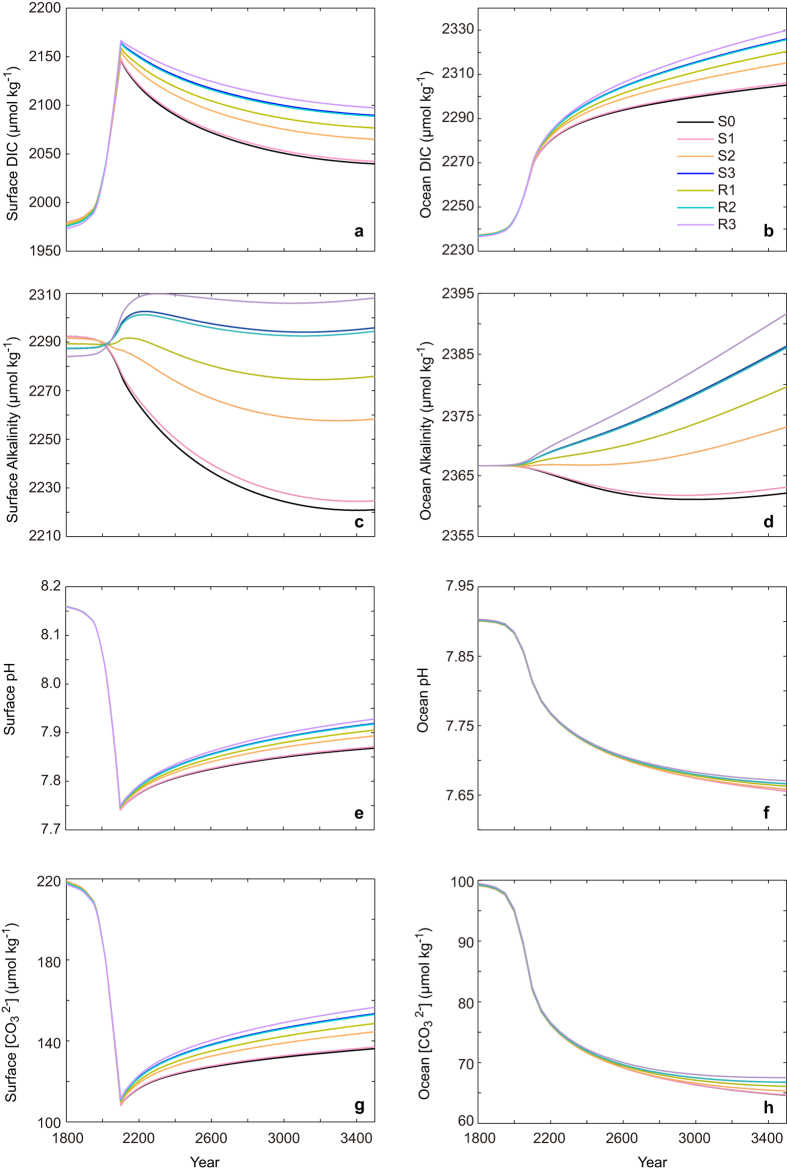
Model-simulated time series of global mean variables as a function of year for (a) ocean surface dissolved inorganic carbon (DIC) concentration, (b) ocean mean DIC concentration, (c) ocean surface alkalinity concentration, (d) ocean mean alkalinity concentration, (e) ocean surface pH, (f) ocean mean pH, (g) ocean surface 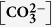, (h) ocean mean 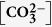. Detailed configuration of different model versions is provided in Table 1.

As shown in Fig. 3, in the control simulation (S0), by year 2100, the global ocean has absorbed 581 PgC of anthropogenic CO_2_. After the cessation of CO_2_ emission at 2100, the ocean continues to absorb CO_2_, and by year 3500, the global ocean has a total CO_2_ uptake of 1462 PgC (Fig. 3b, also see Supplementary Table S1). As for the carbon uptake by the terrestrial biosphere, by year 2100 and 3500 the land has absorbed 629 and 2236 Pg C, respectively (Fig. 3d). Atmospheric CO_2_ reaches a peak value of 897 ppm at year 2100 (Fig. 3e). A cessation of CO_2_ emission leads to a gradual decline of atmospheric CO_2_. By year 3500, atmospheric CO_2_ concentration is 612 ppm with a global mean surface warming of 3.9 °C (Fig. 3f). The ocean’s absorption of anthropogenic CO_2_ acidifies the global ocean (Fig. 4). By year 2100, relative to the preindustrial values, surface mean pH drops by 0.42 units, corresponding to a 50.6% reduction in surface 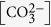. With the gradual decrease of atmospheric CO_2_, surface ocean acidification slowly recovers. By year 3500, surface mean pH reduces by 0.29 units, corresponding to a 37.8% decrease of 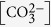 relative to the preindustrial values (see Supplementary Table S1 online).

**Figure 5:**
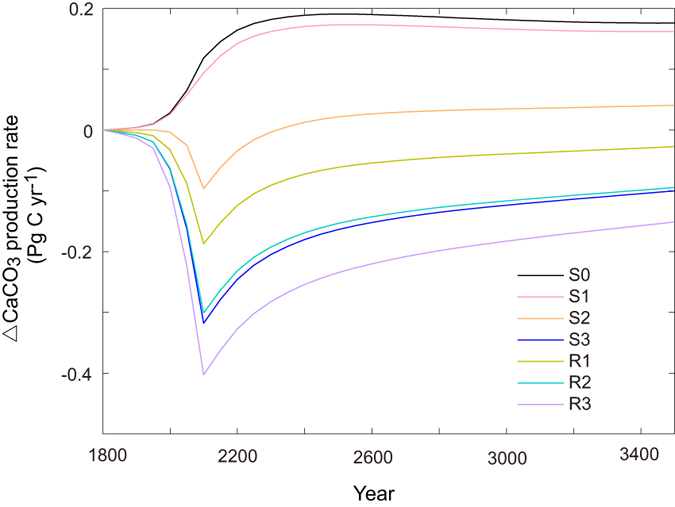
Model-simulated time series of annual and global mean change in CaCO_3_ production rate for different parameterization schemes of CO_2_-calcification feedbacks. Detailed configuration of different model versions is provided in Table 1.

The introduction of 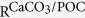 dependence on calcite saturation state 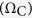 affects the ocean carbon cycle through its impact on the production of CaCO_3_. In the S0 simulation, CaCO_3_ production increases with time (Fig. 5) mainly as a result of increasing ocean temperature that boosts the growth of phytoplankton, which CaCO_3_ production depends on according to equation (2). In the simulations with 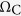-dependent 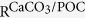, CaCO_3_ productions are under the influence of both changing temperature and 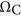. As shown in Fig. 5, except for S1, relative to the preindustrial value, there is a general decrease of CaCO_3_ production with time, indicating the dominant influence of decreasing 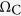. After around year 2150, there appears to be a recovery of CaCO_3_ production as a result of the recovery of surface 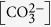 (Fig. 5). In the simulation of S2, after year 2350, the change of CaCO_3_ production becomes positive again, indicating the increasing influence of rising ocean temperature. Overall, compared to the simulation of S0, the introduction of 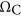-dependent 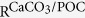 greatly decreases the production of CaCO_3_, which has great implication for the oceanic uptake of CO_2_ as discussed below.

**Figure 6:**
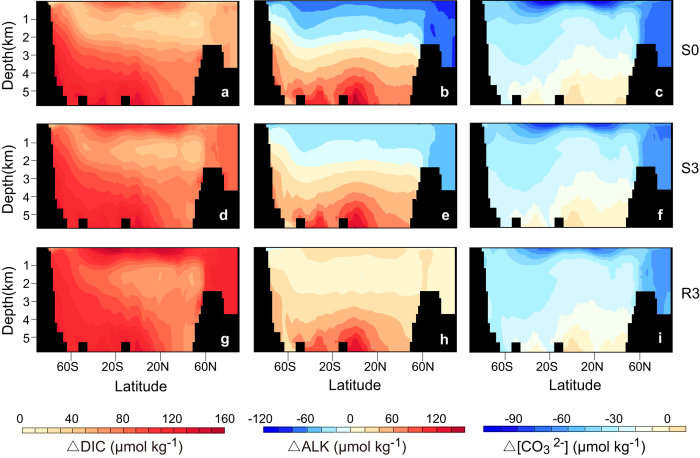
Model-simulated latitude-depth distribution of the change in DIC (year 3500 minus year 1800) ∆DIC (a,d,g), the change in ALK (year 3500 minus year 1800) ∆ALK (b,e,h), and the change in 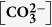 (year 3500 minus year 1800) ∆ 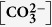 (c,f,i). Results are shown for simulation S0 (**a**–**c**), simulation S3 (**d**–**f**), and simulation R3 (**g**–**i**), respectively. Detailed configuration of different model versions is provided in Table 1. The figures were generated using UV-CDAT (http://uvcdat.llnl.gov/).

The change in CaCO_3_ production has great impact on the ocean alkalinity. In the simulation of S0, an increase in CaCO_3_ production (Fig. 5) leads to a decrease in ocean-mean alkalinity (Fig. 4). In the simulations with the 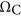-dependent 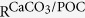, ocean-mean alkalinity generally increases with time. For example, in the simulation of R3, by year 3500, ocean-mean and surface-mean alkalinity have increased by 25 and 24 μmol kg^−1^ respectively (see Supplementary Table S1 online). Meanwhile, in the simulations that include the 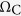-dependent 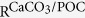, the vertical gradient of alkalinity diminishes due to the reduced CaCO_3_ production rate and the consequent weaker CaCO_3_ pump (Fig. 6, see Supplementary Fig. S1 online).

As a result of modification of the ocean alkalinity, the 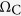-dependent 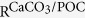 affects the ocean’s uptake of CO_2_. By year 2100, compared to the S0 simulation, the inclusion of 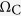-dependent 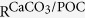 increases accumulated oceanic CO_2_ uptake by 1 PgC (0.1%) to 36 PgC (6.2%), depending on the exact form of CaCO_3_ production parameterization (Fig. 3). By year 3500, the increase in accumulated oceanic CO_2_ uptake relative to the simulation of S0 ranges from 9 PgC (0.6%) to 285 PgC (19.5%) across different CaCO_3_ production parameterization schemes (Fig. 3, see Supplementary Table S1 online). As a consequence, by year 3500, simulated atmospheric CO_2_ ranges from 608 to 542 ppm with the inclusion of dependence of 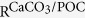 on 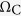, compared with 612 ppm in the S0 simulation (Fig. 3). Moreover, by year 3500, the inclusion of 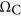-dependent 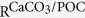 acts to reduce the amount of surface warming by 0.04 to 0.6 K relative to the S0 simulation, depending on the 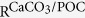 parameterization scheme used (see Supplementary Table S1 online).

The inclusion of 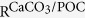 dependence on 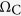 also has a great influence on ocean acidification (Fig. 4 e, f, g, h). For example, by year 3500, the difference of surface mean pH between R3 and S0 becomes 0.06 units, corresponding to a 15% difference of 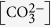 (see Supplementary Table S1 online). Thus, the inclusion of 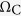-dependent 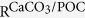 increases the ocean uptake of CO_2_, but mitigates ocean acidification. The mitigation of ocean acidification in the simulations with 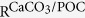 dependence of 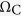, relative to the simulation with fixed 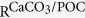 is mainly a result of increased alkalinity, which dominates the effect of increased DIC on ocean acidification.

**Figure 7:**
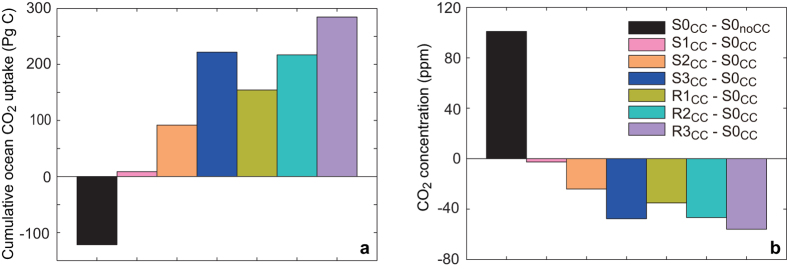
Simulated effects of different CO_2_-calcification feedback parameterization schemes (colored bars) compared with the effect of CO_2_-induced warming (black bars) in year 3500 for (a) cumulative ocean CO_2_ uptake, (b) atmospheric CO_2_ concentration. The effect of CO_2_-calcification feedback is represented by the difference between S1 (S2, S3, R1, R2, R3) simulation with the inclusion of CO_2_-induced warming and the S0 simulation with the inclusion of CO_2_-induced warming. The effect of CO_2_-induced warming is represented by the difference between S0 simulation with the inclusion of CO_2_-induced warming and the S0 simulation without it. Detailed configuration of different model versions is provided in Table 1.

In the above, we have discussed model-simulated results with the inclusion of CO_2_-induced warming. To test the importance of CO_2_-induced warming on the ocean carbon cycle, we have performed additional simulations that do not include the radiative effect of increasing atmospheric CO_2_. Our simulations show that, by year 3500, in the S0 case, in the absence of CO_2_-induced warming effect, model-simulated cumulative ocean’s uptake of CO_2_ is 122 PgC greater than that in the simulation with CO_2_-induced warming (Fig. 7, see Supplementary Fig. S2, Supplementary Table S1 online). For comparison, relative to the S0 simulation, by year 3500, the inclusion of 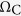-dependent 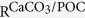 increases the ocean’s uptake of CO_2_ by 9 to 285 PgC (Fig. 7, see Supplementary Fig. S2, Supplementary Table S1 online). This comparison shows that in terms of the magnitude of oceanic CO_2_ uptake, the effect of CO_2_-calcification feedback could be comparable to or even much larger than that from the feedback of CO_2_-induced warming.

## Discussion

Here, we use the UVic model to quantify the effect of potential CO_2_-calcification feedback on the projections of the ocean carbon cycle and climate change. To evaluate the effect of CO_2_-calcification feedback on the ocean carbon cycle and associated uncertainties, we include two different types of parameterization schemes that link CaCO_3_ production with saturation state of calcite. In each scheme, a set of different parameters is used. As atmospheric CO_2_ increases and the ocean becomes more acidic, the introduction of 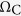-dependent 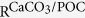 decreases the production of CaCO_3_, increasing ocean alkalinity and enhancing the oceanic uptake of atmospheric CO_2_. Therefore, it triggers negative feedbacks on the growth of atmospheric CO_2_ and curbs global warming to a certain degree. Under SRES A2 CO_2_ emission scenario with zero emission after year 2100 and a total cumulative emission of 2270 PgC, relative to the simulation with fixed CaCO_3_: POC production ratio, by year 2100, the simulations that include CO_2_-calcification feedback decrease modeled atmospheric CO_2_ by 0.1 to 7 ppm; by year 3500, the simulations that include CO_2_-calcification feedback decrease modeled atmospheric CO_2_ concentration by 4 to 70 ppm. The magnitude of the CO_2_-calcification feedback depends on the calcification-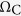 parameterization scheme used and parameter values used, demonstrating the importance of both the model structure uncertainty and parameter uncertainty of the CO_2_-calcification feedback in regulating the ocean carbon cycle. While the inclusion of the CO_2_-calcification feedback enhances the ocean’s uptake of atmospheric CO_2_, it acts to mitigate ocean acidification mainly as a result of increased ocean alkalinity. For example, by year 3500, the inclusion of 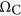-dependent 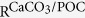 increases surface mean pH and 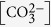 by 0.8% and 15.0% in R3 relative to S0. Furthermore, our simulations show that the effect of CO_2_-calcification feedback on ocean’s uptake of atmospheric CO_2_ is comparable to, and in some cases, much larger than the effect from CO_2_-induced warming.

Our study shows a noticeable CO_2_-calcification feedback on atmospheric CO_2_. Different estimates of this feedback are reported in previous modeling studies^21^,^22^,^23^,^24^. For example, Ridgwell *et al.* by using the function form of equation (4) to represent CO_2_-calcification feedback, reported that under a CO_2_ emission scenario that reaches a total of 4,000 PgC, the inclusion of CO_2_-calcification feedback would lower atmospheric CO_2_ concentration by 29–93 ppm by year 3000, depending on the parameter values used^21^. Hofmann and Schellnhuber^22^, using an exponential form that links calcification rate to carbonate ion concentration, predicted a 125 ppm decrease in atmospheric CO_2_ by year 3000 as a result of CO_2_-calcification feedback under a scenario with total anthropogenic CO_2_ emission of 4,075 PgC. Other studies show relatively small effects from the CO_2_-calcification feedback. For instance, Heinze, using a linear relationship that links calcification to seawater CO_2_ partial pressure, projected a CO_2_ decrease of 12 ppm due to CO_2_-calcification feedback when atmospheric CO_2_ reaches about 1400 ppm^23^; Gangstø *et al*.^24^, by using both Michaelis-Menten type formula and linear relationship to link calcification and CaCO_3_ saturation, showed that by year 2100, the CO_2_-calcification feedback acts to reduce atmospheric CO_2_ concentration by 1 to 11 ppm under a range of parameterization schemes and IPCC scenarios of RCP8.5 and RCP6.0. Although these studies, including our study here, are not directly comparable because of different CO_2_ pathways used and different inherent model structures in ocean dynamics and biogeochemistry, at least part of the difference is associated with different representations of CO_2_-calcification feedback. Uncertainties in our model results here reflect uncertainties in modeled parameterization of CO_2_-calcification feedback, which actually reflects uncertainties in our understanding of the calcification response to changing ocean chemistry. The reported response of the CaCO_3_ production rate to ocean acidification varies dramatically between experiments using different species of calcifying groups or manipulation methods^20^,^26^. Therefore, modeling simulations based on different results of experimental studies would result in different estimates of the effect of CO_2_-calcification feedback. More coordinated experimental and observational studies on the CaCO_3_ production response to ocean acidification are needed for a more reliable appraisal of the CO_2_-calcification feedback.

In this study, we have investigated the response of calcification to acidification and its feedback to the ocean carbon cycle. Other processes relevant to CaCO_3_ cycle that are not included in this study could also have important effect on the ocean carbon cycle. For example, inclusion of the dependence of CaCO_3_ dissolution rate on CaCO_3_ saturation state would further alter the ocean carbon cycle^19^,^27^. In addition, the ballast effect, i.e., the link between the fluxes of particulate organic carbon (POC) and particulate inorganic carbon (PIC) to the abyssal ocean^28^,^29^, is not included in the model. It is possible that reduced CaCO_3_ production could result in a decrease in PIC export rate, which consequently lowers POC export rate. This reduced vertical transport of POC would weaken the oceanic carbon pump and decrease the capacity for the global ocean to absorb atmospheric CO_2_, acting as a positive feedback to atmospheric CO_2_^22^,^30^. The feedback from the ballast effect could partly counteract the CO_2_-calcification feedback, which merits further study.

This study demonstrates the potential important effect of CO_2_-calcification feedback on the ocean carbon cycle and atmospheric CO_2_ on the timescale from centuries to millennia. Further experimental and modeling studies are needed to acquire a better understanding of the CO_2_-calcification feedback, which is crucial for a reliable projection of future atmospheric CO_2_ concentrations and climate change.

## Methods

### Model description

An Earth system model of intermediate complexity, UVic ESCM (the University of Victoria Earth System Climate Model) version 2.9^31^, was used for this study. UVic consists of a 3D ocean general circulation model (Modular Ocean Model 2 or MOM2) with a resolution of 1.8° latitude by 3.6° longitude and 19 vertical layers in the ocean. The ocean component is coupled to a vertically integrated energy-moisture balance atmospheric model and a thermodynamic/dynamic sea ice model^32^. The land carbon cycle component is represented by a dynamic vegetation model (the Hadley Center model TRIFFID) and a land surface model (the Met Office surface exchange scheme or MOSES)^33^. The ocean carbon cycle is represented by an inorganic carbon cycle model following the protocol of the Ocean Carbon-Cycle Model Intercomparison Project (OCMIP)^34^, and a nutrient-phytoplankton-zooplankton-detritus (NPZD) marine ecosystem model^31^. The ocean carbon cycle model also comprises a sediment component that calculates CaCO_3_ concentration from CaCO_3_ dissolution and burial rates^35^,^36^.

The CaCO_3_ production in the model is calculated as


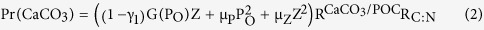


where Pr(CaCO_3_) represents CaCO_3_ production, 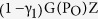 denotes the zooplankton (Z) grazing of phytoplankton 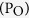, 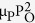 represents the mortality of phytoplankton, 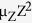 denotes the mortality of zooplankton, 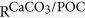 represents the ratio of CaCO_3_ production to the production of particulate organic carbon, and 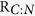 is the carbon to nitrogen Redfield ratio^31^.

### Parameterization of CaCO_3_ production

In the original model, CaCO_3_: POC production ratio 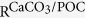 in equation (2) is fixed at a constant value of 0.018. In this study, two types of parameterization functions of 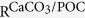 that link CaCO_3_ production with saturation state of calcite 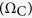 are introduced into the UVic model.

The first type of parameterization follows the Michaelis-Menten function based on Pinsonneault *et al.*^37^:


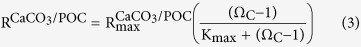


Where 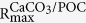 denotes the specified maximum value of 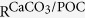 (the CaCO_3_: POC production ratio), and 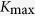 is a half-saturation constant^37^,^38^. Different model versions based on this parameterization are denoted as “series S” (Table 1). In series S, the values of 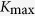 are selected to be 0.07, 1.5 and 20, which covers the range of 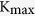 values used by Pinsonneault *et al.*^37^. For each 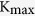, we calculate the corresponding value of 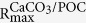 by using the modeled preindustrial global mean sea surface calcite saturation state 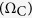 of 5.2 to ensure that the preindustrial surface mean CaCO_3_: POC production ratio in each model version has the same value of 0.018, i.e., the fixed constant used in the original model.

The second type of parameterization follows the thermodynamically-based function based on Ridgwell *et al.*^21^,^39^:


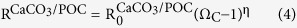


Where 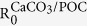 represents a spatially-uniform constant and 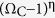 is a thermodynamically-based modifier with the power parameter η^21^,^39^. Different model versions based on this parameterization are denoted as “series R” (Table 1). In series R, we set the values of η to be 0.53, 0.81, and 1.09, which covers the range of η values used by Ridgwell *et al.*^21^, and for each η we obtain the value of 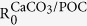 in the same way as 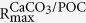 in series S.

All together, we have a set of model versions with different parameterizations of 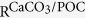 (S1 to S3 and R1 to R3 in Table 1). In addition, we have the original model configuration with fixed 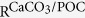 (S0 in Table 1). For each parameterization, the dependence of CaCO_3_: POC production ratio on 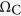 are presented in Fig. 1.

### Simulation experiments

All of the model versions mentioned above are integrated for 10,000 model years with fixed preindustrial atmospheric CO_2_ concentration of 280 ppm to reach a quasi-equilibrium preindustrial state of global climate and carbon cycle. Using the preindustrial climate state as initial condition for the nominal year of 1800, two sets of 1700-year transient simulations are performed (from year 1800 to 3500). The first set of simulation includes the feedback from CO_2_-induced warming on the ocean carbon cycle, whereas the second set of simulation does not include the radiative effect of increasing CO_2_ on global climate. Each set of experiments includes seven simulations, corresponding to the seven model versions listed in Table 1. All these 14 simulations are integrated under the IPCC CO_2_ emission scenario SRES A2 (“business-as-usual”) until year 2100. After year 2100, emissions are set to zero. During this spinup stage, at each time step continental weathering CaCO_3_ flux via river discharge is set equal to the model-simulated accumulation flux of CaCO_3_ from open ocean to deep-sea sediment. For transient simulations, weathering flux is held fixed at the rate that is diagnosed from the spinup run, whereas CaCO_3_ accumulation flux is allowed to evolve freely.

## Additional Information

**How to cite this article**: Zhang, H. and Cao, L. Simulated effect of calcification feedback on atmospheric CO_2_ and ocean acidification. *Sci. Rep.* **6**, 20284; doi: 10.1038/srep20284 (2016).

## Supplementary Material

Supplementary Information

## Figures and Tables

**Table 1:** Summary of configurations of different model versions with different parameterizations of the dependence of calcification rate on saturation state of CaCO_3_.

Michaelis-Menten Function: 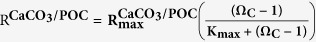				
Model Version	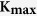	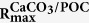	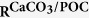 at Preindustrial Surface 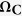	Preindustrial Surface 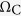
S1	0.07	0.0183	0.0180	5.2
S2	1.5	0.0244	0.0180	5.2
S3	20	0.1037	0.0180	5.2
**Thermodynamically-based Function:** 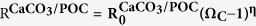				
**Model Version**	**η**	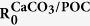	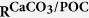 **at Preindustrial Surface** 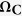	**Preindustrial Surface** 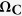
R1	0.53	0.0084	0.0180	5.2
R2	0.81	0.0056	0.0180	5.2
R3	1.09	0.0038	0.0180	5.2
Control Simulation				
S0	—	—	0.0180	5.2
