# Supplementary material for: Simulated effect of calcification feedback on atmospheric CO2 and ocean acidification
Source: Sci Rep. 2016 Feb 3;6:20284. doi: 10.1038/srep20284 (PMC4738325; doi:10.1038/srep20284)
Supplement: Supplementary Information [file srep20284-s1.pdf]

Simulated effect of calcification feedback on atmospheric CO_2 and ocean acidification

Han Zhang[1] and Long Cao[1,2,*]

[1] School of Earth Sciences, Zhejiang University, Hangzhou, Zhejiang 310027, China

[2] State Key Laboratory of Satellite Ocean Environment Dynamics, Second Institute of Oceanography, Hangzhou, Zhejiang, China

*Email: longcao@zju.edu.cn

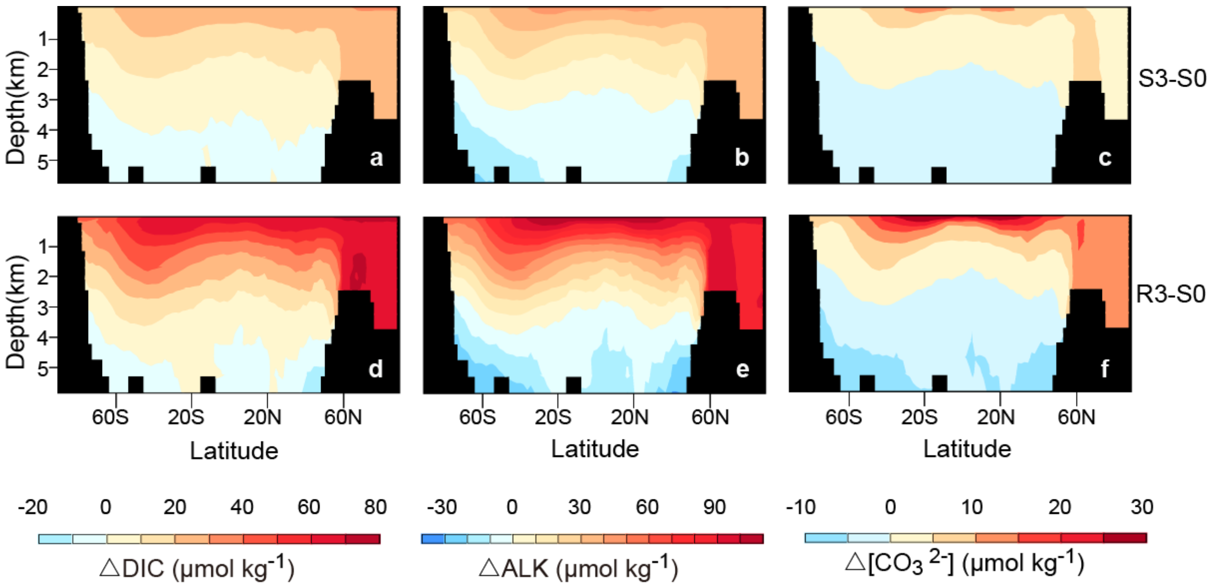

Supplementary Figure S1. The difference in model-simulated latitude-depth distribution of (a, d) ΔDIC, (b, e) ΔALK, (c, f) $\Delta[CO_3^{2-}]$. Results are shown for the difference between simulation S3 and S0 (a, b, c) and simulation R3 and S0 (d, e, f), respectively. The figures were generated using UV-CDAT (http://uvcdat.llnl.gov/). Detailed configuration of different model versions is provided in Table 1.

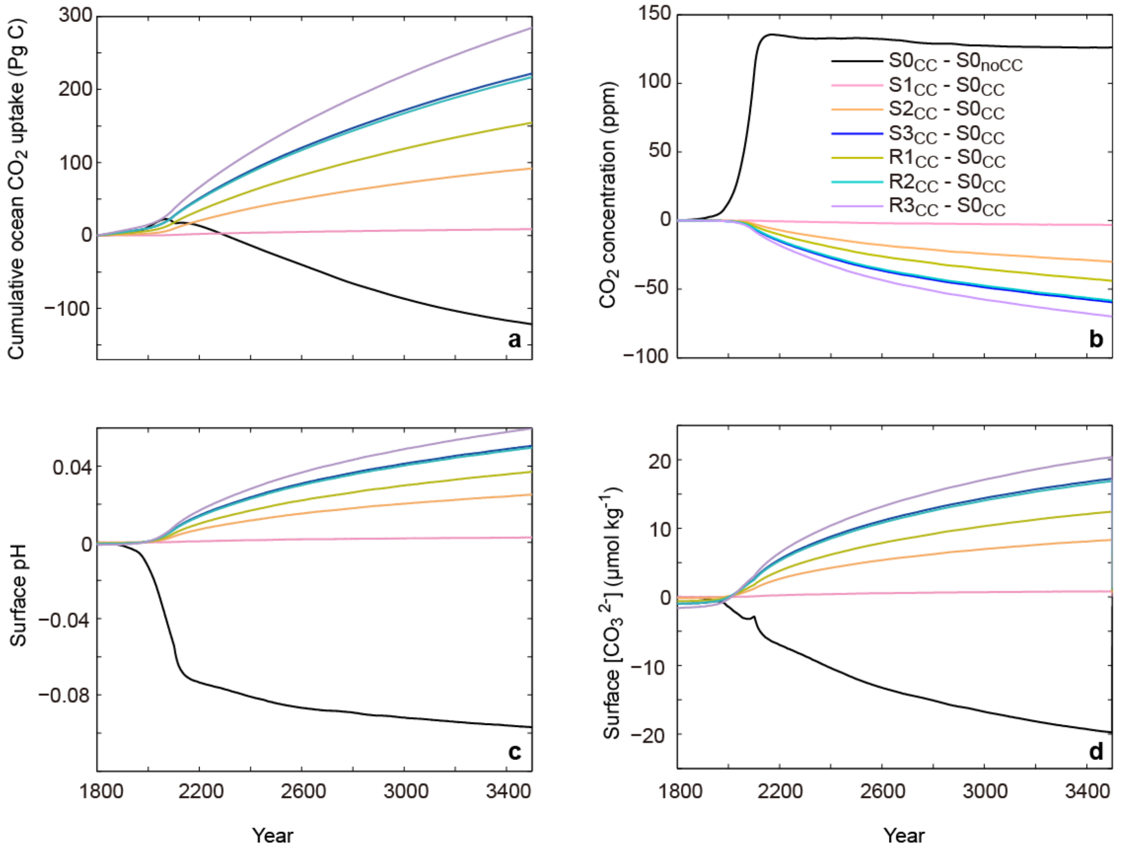

Supplementary Figure S2. Simulated effects of different CO_2-calcification feedback parameterization schemes (colored lines) compared with the effect of CO_2-induced warming (black lines) for (a) cumulative ocean CO_2 uptake, (b) atmospheric CO_2 concentration, (c) ocean surface pH, (d) ocean surface $[CO_3^{2-}]$. The effect of CO_2-calcification feedback is represented by the difference between S1 (S2, S3, R1, R2, R3) simulation with the inclusion of CO_2-induced warming and the S0 simulation with the inclusion of CO_2-induced warming. The effect of CO_2-induced warming is represented by the difference between S0 simulation with the inclusion of CO_2-induced warming and the S0 simulation without it. Detailed configuration of different model versions is provided in Table 1.

	S0	S1	S2	S3	R1	R2	R3
Δ $CaCO_3$ production rate (PgC yr^{-1})	0.176/0.013	0.162/0.006	0.041/-0.063	-0.100/-0.163	-0.027/-0.112	-0.095/-0.160	-0.151/-0.204
Cumulative ocean CO_2 uptake (PgC)	1462/1584	1471/1588	1554/1642	1684/1749	1616/1694	1679/1745	1747/1806
Δ atmospheric CO_2 (ppm)	331.1/205.0	327.8/204.1	301.1/194.6	271.6/181.3	287.2/188.2	272.7/181.8	261.2/175.9
Δ sea surface alkalinity (μmol kg^{-1})	-71.4/7.6	-67.7/9.2	-33.3/26.6	8.5/53.4	-13.4/39.7	7.0/52.6	24.1/64.7
Δ ocean mean alkalinity (μmol kg^{-1})	-4.5/17.4	-3.5/17.9	6.4/23.9	19.7/34.1	12.9/28.9	19.4/33.8	25.0/38.8
Δ sea surface DIC (μmol kg^{-1})	60.6/109.1	63.1/110.1	86.3/121.4	114.3/138.8	99.8/130.0	113.3/138.4	124.6/146.1
Δ ocean mean DIC (μmol kg^{-1})	67.9/86.2	68.9/86.7	77.8/91.8	89.0/100.1	83.2/95.8	88.7/99.8	93.3/103.7
Δ sea surface pH	-0.293/-0.196	-0.290/-0.195	-0.268/-0.185	-0.241/-0.170	-0.255/-0.177	-0.242/-0.170	-0.232/-0.164
Δ ocean mean pH	-0.245/-0.202	-0.245/-0.202	-0.241/-0.200	-0.235/-0.196	-0.238/-0.198	-0.235/-0.196	-0.235/-0.193
Δ sea surface [CO_3^{2-}] (μmol kg^{-1})	-82.9/-63.1	-82.1/-62.8	-74.4/-58.4	-64.7/-51.7	-69.9/-55.2	-65.0/-51.9	-60.9/-48.8
Δocean mean [CO_3^{2-}] (μmol kg^{-1})	-34.4/-33.2	-34.3/-33.2	-33.6/-32.6	-32.3/-31.4	-32.9/-32.0	-32.3/-31.4	-31.7/-30.8

Supplementary Table S1. Simulated ocean chemistry change (relative to year 1800) at year 3500 for the simulations with (first numbers) and without (second numbers) the effect of CO_2-induced warming on the ocean carbon cycle.
